# A First Insight into the Gut Microbiota of the Sea Turtle *Caretta caretta*

**DOI:** 10.3389/fmicb.2016.01060

**Published:** 2016-07-07

**Authors:** Khaled F. A. Abdelrhman, Giovanni Bacci, Cecilia Mancusi, Alessio Mengoni, Fabrizio Serena, Alberto Ugolini

**Affiliations:** ^1^Dipartimento di Biologia, Università di FirenzeSesto Fiorentino, Italy; ^2^Agenzia Regionale per la Protezione Ambientale della ToscanaLivorno, Italy

**Keywords:** microbial communities, gut, Loggerhead Turtle, *Caretta caretta*, 16S rRNA gene, microbiome, *Vagoccoccus*

## Introduction

In the last years the microbial communities (microbiota) associated with the digestive tract of animals have been subjected to wide research interest (Ley et al., 2008; Zhu et al., 2010; Huttenhower et al., 2012). The presence of functional relationship between the host and the associated microbiome (the genes and genomes of the microbiota) has been highlighted, and the new term of hologenome has been proposed to refer to the set of functions (genes) of host and microorganisms associated with it (Zilber-Rosenberg and Rosenberg, 2008). The study of model animals has revealed roles for the microbiome in adaptive immunity development and in host physiology, ranging from mate selection to skeletal biology and lipid metabolism (Ley et al., 2008; Kostic et al., 2013; Du Toit, 2016). For vertebrates, most of the studies on gut microbiota and microbiome have been performed in mammals (i.e., mouse, rat and humans) and in fishes (as the model *Danio rerio*) (Huttenhower et al., 2012; Kostic et al., 2013). Recently, microbiotas and microbiomes of non-model organisms have started to be investigated with the aim to shed light on animal-associated microbial diversity (Keenan et al., 2013; Mengoni et al., 2013; Cahill et al., 2016) and to potentially discover new biotechnologically important microbial strains (Papaleo et al., 2012; Sanchez et al., 2012).

Sea turtles (*Testudines, Reptilia*) occur in oceanic and neritic habitats, from the tropics to subarctic waters, and venture onto terrestrial habitats to nest or bask in tropical and temperate latitudes. Sea turtle populations around the world have dwindled and, in many places, continue to decline (Wallace et al., 2010). *Caretta caretta L*. (Loggerhead Turtle) is distributed throughout the subtropical and temperate regions of the Mediterranean Sea and Pacific, Indian, and Atlantic Oceans. Loggerhead Turtle is classified as Vulnerable A2b in the IUCN Red List (http://www.iucnredlist.org/details/3897/0). The Loggerhead Turtle plays important roles in maintaining marine ecosystem (Bjorndal and Jackson, 2002; Bolten and Witherington, 2003). These roles range from maintaining productive coral reef ecosystems to transporting essential nutrients from the oceans to beaches and coastal dunes. However, in spite of the considerable importance for the study of vertebrates, few studies only are present on microbial communities associated with sea turtles (Ferronato et al., 2009; Sarmiento-Ramírez et al., 2014; Yuan et al., 2015) and no reports on gut microbial communities.

The aim of this work is the characterization, for the first time, of the gut microbiota of the sea turtle *C. caretta*, to shed a preliminary light on its features with respect to other reptiles and to marine vertebrates. Both feces and intestine samples were taken to have the wider overview of gut microbiota taxonomic composition.

## Links to deposited data

The sequences dataset (Table 1) was deposited in the GenBank database (URL: http://www.ncbi.nlm.nih.gov/bioproject/; Bioproject PRJNA314462, Biosample accessions SAMN04508196-SAMN04508205). Users can download and use the data freely for research purpose only with acknowledgment to us and quoting this paper as reference to the data.

**Table 1:** **Samples details and sequencing statistics**.

**Sample code**	**Sample type**	**Sample name**	**Sex**	Dimension^*^	**Days of hospitalization before sampling**	**Sampling date**	Sampling location^**^	**Total Reads**	**Reads Passing Quality Filtering**	**% Reads Passing Quality Filtering**
T1	Faeces	GoGo Luce	Female	37	40	2014-09-30	42.40 N 11.29 E	544605	507072	93.1 %
T3	Faeces	GoGo Luce	Female	37	37	2014-09-27	42.40 N 11.29 E	386371	357231	92.5 %
T4	Intestine	Camilla	Undetermined	52	21	2014-09-13	43.54 N 10.31 E	267169	249587	93.4 %
T5	Intestine	Camilla	Undetermined	52	21	2014-09-13	43.54 N 10.31 E	100635	91047	90.5 %
T6	Intestine	RT46CC/2014	Undetermined	47	0 (death, under decomposition)	2014-07-29	43.54 N 10.31 E	40231	37117	92.3 %
T7	Intestine	RT44CC	Undetermined	65	0 (death recently)	2014-07-21	43.54 N 10.31 E	220358	207119	94.0 %
T9	Intestine	RT51CC	Undetermined	56	0 (death recently)	2014-09-12	43.54 N 10.31 E	116819	108915	93.2 %
T10	Intestine	Genova	Female	52	22	2014-09-13	44.41 N 8.92 E	129634	111712	86.2 %
T11	Faeces	F2600_Ondina	Female	54	28	2015-02-25	42.40 N 11.29 E	115155	109961	95.5 %
T12	Faeces	GR001_Olivia	Female	63	41	2015-03-09	42.40 N 11.29 E	108224	102629	94.8 %

* *The length of the standard curve in cm is reported*.

** *The location of the collection is that of the recovery center*.

## Materials and methods

### Sampling and sequence production

Samples of feces and intestine of *C. caretta* were collected in the years 2014 and 2015, from different individuals stranded or accidentally caught along the Tyrrhenian sea coast in Tuscany and Liguria regions (Italy). Animals were hosted in the recovery centers associated with network of the Tuscan Observatory for Biodiversity. A total 10 samples of eight individuals was analyzed (Table 1). The samples consisted of four samples of feces (T1, T3, T11, T12) and six cloacal contents and intestine sections (colorectal) (T4, T5, T6, T7, T9, T10). Intestine sections were collected from animals stranded or dead in the recovery centers, immediately after retrieval. Faeces were collected immediately after deposition from living animals in hospitalized conditions in the recovery centers. T1 and T3 were feces from the same individual (“GoGo Luce”) collected in different days (at 37 and 40 days after hospitalization), as well as T4 and T5 were different portions of cloacal samples from the same individual (“Camilla”). All samples were immediately stored at −20°C prior of the extraction of DNA.

DNA was extracted, simultaneously for all samples, from feces, cloacal contents and gut tissues using the FastDNA™ SPIN Kit for soil (MP Biomedicals, Italy). From the extracted DNA, the bacterial V4 region of 16S rRNA genes was amplified with specific primers (515F, 806R) using the protocol reported in the 16S Metagenomic Sequencing Library Preparation protocol from Illumina (Part # 15044223 Rev. B; URL: http://www.illumina.com/content/dam/illumina-support/documents/documentation/chemistry_documentation/16s/16s-metagenomic-libraary-prep-guide-15044223-b.pdf). PCR products were sequenced in a single run using Illumina MiSeq technology with pair-end sequencing strategy with MiSeq Reagent Kit v3. Library preparation and demultiplexing have been performed following Illumina 's standard pipeline (Caporaso et al., 2012).

### Raw data processing and statistical analyses

Raw sequences were clustered into “Operation Taxonomic Units” (OTUs) following the UPARSE pipeline as previously described (Bacci et al., 2015a,b). A pre-processing step was also included using StreamingTrim (Bacci et al., 2014), to remove low-quality reads that can generate errors in downstream analyses. Read pairs were merged using PANDAseq assembler with default settings (Masella et al., 2012). Singletons were removed before the OTU clustering step, which was performed using an identity threshold of 97%. Chimeras were detected and removed by UPARSE during clustering step (“cluster_otus” command). Finally, from OTU cluster, a single representative sequence was selected and used for taxonomical identification by SINA classifier on the latest SILVA dataset available when we performed the analysis (SSURef Nr99 version 119). Reads which were attributed to chloroplast and mithocondria were removed from the OTU table. All steps were implemented with an in-house pipeline available at (https://github.com/GiBacci/o2tab).

Collected 16S rRNA sequences were taxonomically classified using the Ribosomal Database Project classifier with 80% confidence threshold, as the most informative threshold (Masella et al., 2012).

Rarefaction analysis was carried out plotting the number of observed OTUs against the number of reads at genus level (Table S1). Tabulated values were used to produce a rarefaction curve for each sample and estimate diversity values. Specific differences in community composition and the similarity among microbial communities was determined using similarity percentage (simper) analysis and Principal Component Analysis (PCA). Both analyses were performed with the modules present in PAST (PAlaeontological STatistics) ver. 3 software (Hammer et al., 2001).

### Ethical statement

Samples were collected from hospitalized animals (the feces) or dead animals (the intestine samples). All animals were kept in Authorized Recovery Centers (as defined by the Italian regulation).

## Results

A total 1882390 reads of all samples of *C. caretta* passed quality filtering sequences (92.8% of total reads) (Table S1). After OTU assignment (Table S1) rarefaction curves obtained reached or nearly reached a plateau, indicating a satisfactory level of diversity sampling (Figure S1).

**Figure 1:**
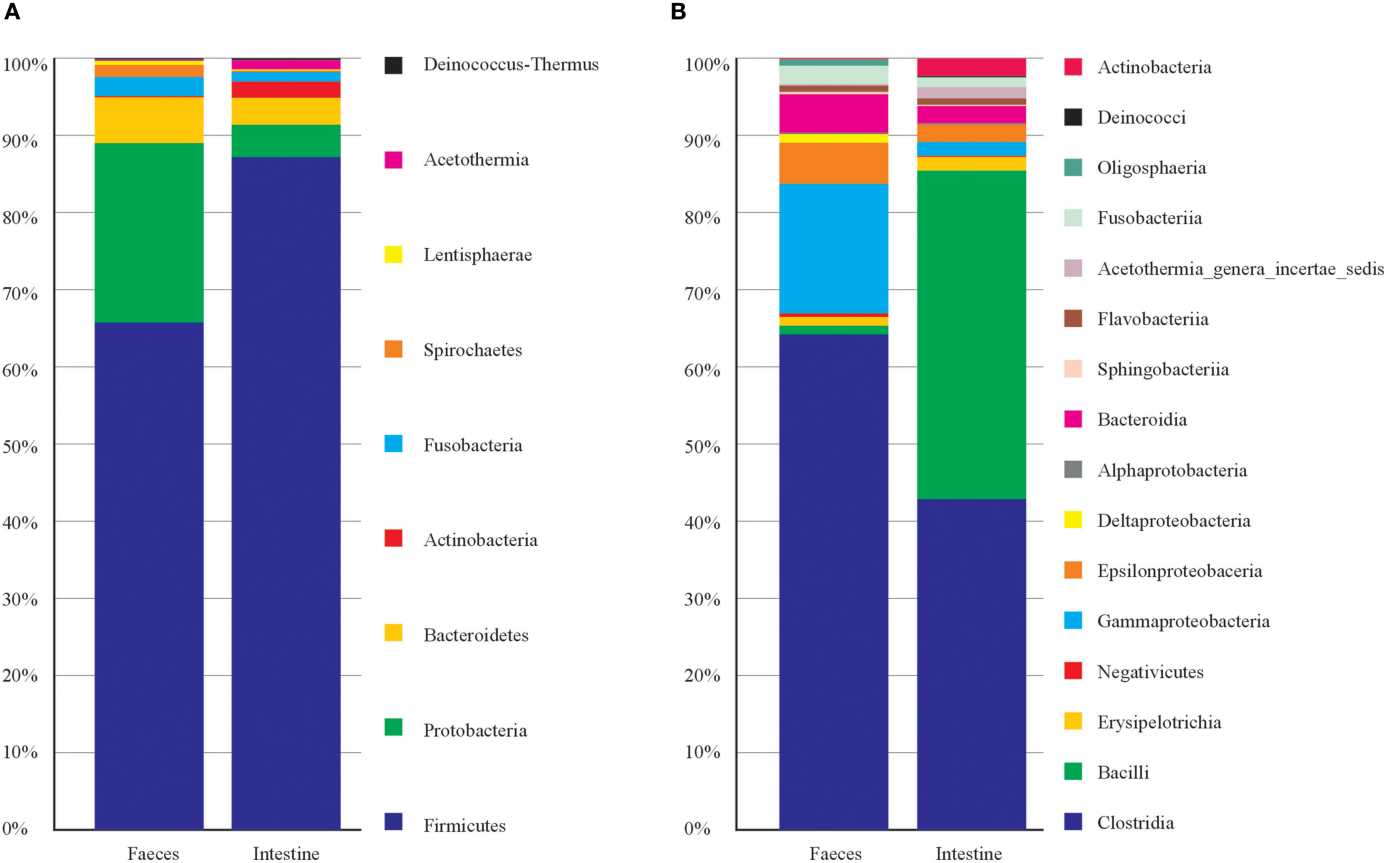
**Taxonomic composition of ***C. caretta*** gut and faeces microbiota at different taxonomic levels**. The percentage of occurrence of each taxon is reported as cumulative bar chart. **(A)** Phylum; **(B)** Class. The legend shows the list of taxa from top to bottom of the bars.

Concerning the taxonomic composition (Figure 1) feces samples were dominated by members of phyla *Firmicutes* (66%), *Proteobacteria* (23%), *Bacteroidetes* (6.2%). Within the phylum *Firmicutes* the class *Clostridia* was the most abundant (63.20%). The intestine samples were dominated by phyla *Firmicutes* (87%), *Proteobacteria* (4.2%) and *Bacteroidetes* (3.4%). *Firmicutes* were represented by member of the classes *Clostridia* (43%) and *Bacilli* (42.5%). This latter was entirely represented (100%) by order *Lactobacillale*s (Table S1). While the most represented bacterial genera among intestine samples were *Vagococcus* with 42.3%, and among feces were Clostridium XI 21.3%, and Clostridium sensu strict 14.6% (Table S1). Principal Component Analysis on OTU representation (Figure S2) showed that most of the sample were very similar each other. However, notably the two samples of feces from the same individual (T1 and T3, taken in different times) were separated from the rest of the samples. In particular, for T1 and T3, OTU 3, OTU 4, and OTU 5 (all attributed to *Clostridiales*) collectively contributed for more than 30% of total variance in the differentiation from the other samples (Table S2). Indeed, T1 and T3 were taken from a young female after few days of hospitalization in the recovery center and may mirror the microbiota of a relatively healthy individual in the wild, while the other samples mainly were from animals kept in the recovery centers for longer times. However, we cannot exclude that T1 and T3 microbiota may represent a phase of rapid changes in gut microbiota due to the change in diet (i.e., artificial feeding in the recovery center), which then may bring to a more stable and homogenous microbiota (present in the other samples) after more days. Sampling of more individuals (healthy) would be needed to clarify this issue.

Finally, we inspected which taxa of the microbiota mostly contribute to differentiate feces vs. intestine. Results obtained after Simper analysis showed that the genera mostly contributing to differences were *Vagococcus* (*Bacilli, Enteroccoaceae*) with 11.92%, *Robinsoniella* (Class Clostridia) with 6.29%, this latter represented more in intestine samples, Clostridium XI (Class Clostridia) with 7.37 % and represented more in feces samples (Table S2).

## Conclusions

This first investigation on the gut microbiota of *C. caretta* showed a pattern of taxa which include well know members colonizing vertebrate guts. In particular the most abundant phyla found (*Firmicutes* and *Bacteroidetes*) are also abundant in the human gut (Ley et al., 2008) as well as in other land vertebrates and reptiles (Costello et al., 2010; Keenan et al., 2013). However, especially in the feces samples, *Gammaproteobacteria* were particularly present (more than 15% of total reads) including member of *Oceanospirillales, Alteromonadaceae, Pseudomonadaceae, Enterobacteriaceae*. Moreover, as suggested by T1 and T3 samples, quite important differences in the microbiota could be detected, which may be related to the influence of hospitalization in most of the sampled animals.

The presented data could be used for comparative analyses of vertebrate gut microbiotas.

## Author contributions

KA performed the experiments. GB helped in data analysis. FS, CM performed sampling. AU conceived the work. AM coordinated the work and drafted the manuscript.

### Conflict of interest statement

The authors declare that the research was conducted in the absence of any commercial or financial relationships that could be construed as a potential conflict of interest.

## Supplementary material

Figure S1 **Rarefaction analysis on sequencing data of *C. caretta* gut microbiota**.

Figure S2 **Principal Component Analysis of OTU abundance in each sample**. The percentage of variance displayed by the first two components is reported.

Table S1 **Occurrence of Operative Taxonomic Units (OTUs)**. The number of reads for each OTU is reported for each sample. The taxonomy of each OTU is also reported.

Table S2 **Results of SIMPER analysis on taxa occurrence**. The taxonomic attribution of OTUs, the percentage of contribution and the cumulative contribution are reported.
