# Supplementary figures and images for: A First Insight into the Gut Microbiota of the Sea Turtle Caretta caretta
Source: Front Microbiol. 2016 Jul 7;7:1060. doi: 10.3389/fmicb.2016.01060 (PMC4935691; doi:10.3389/fmicb.2016.01060)

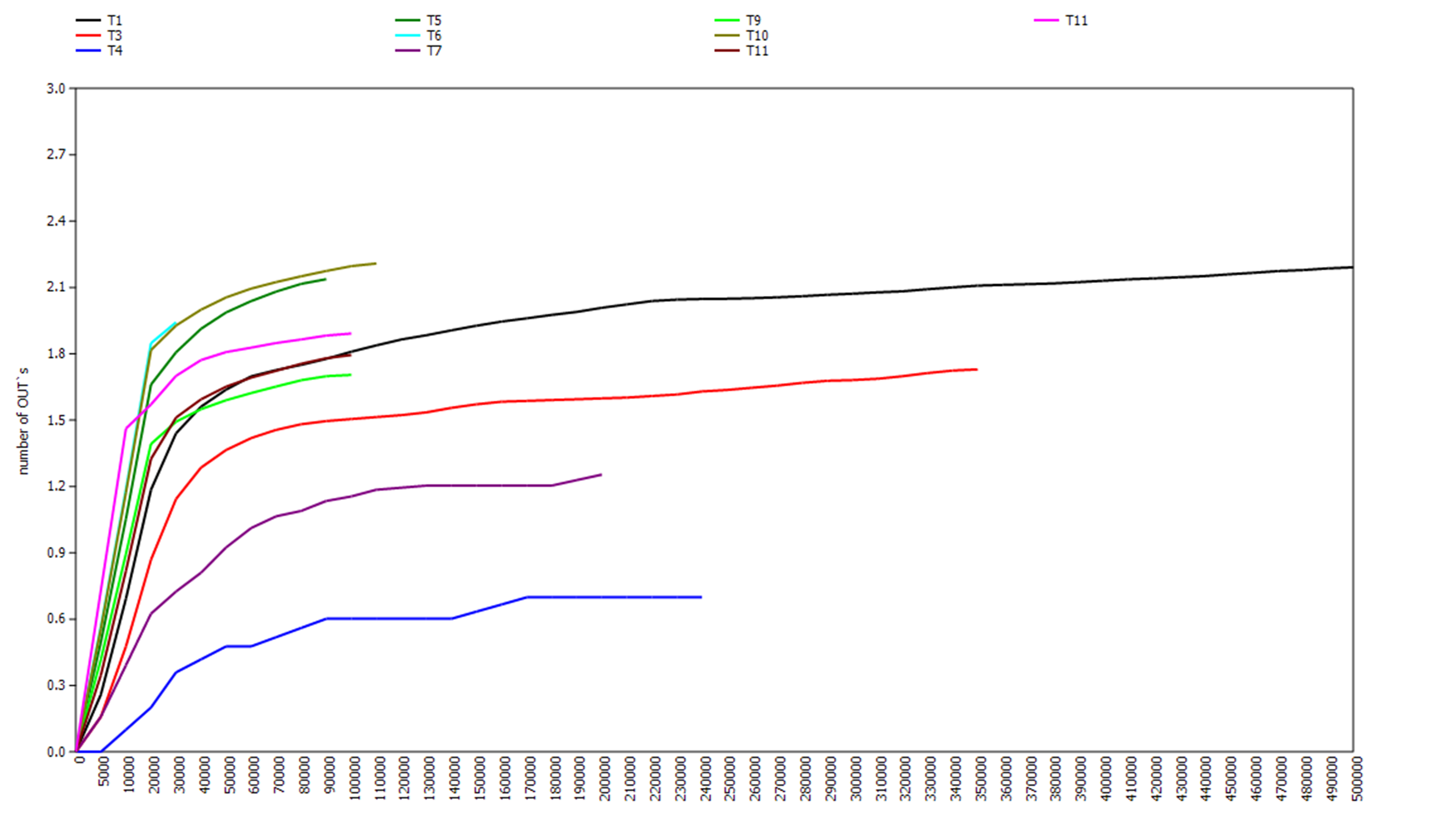

Supplement: Figure S1 — Rarefaction analysis on sequencing data of C. caretta gut microbiota. [file Image1.TIF]

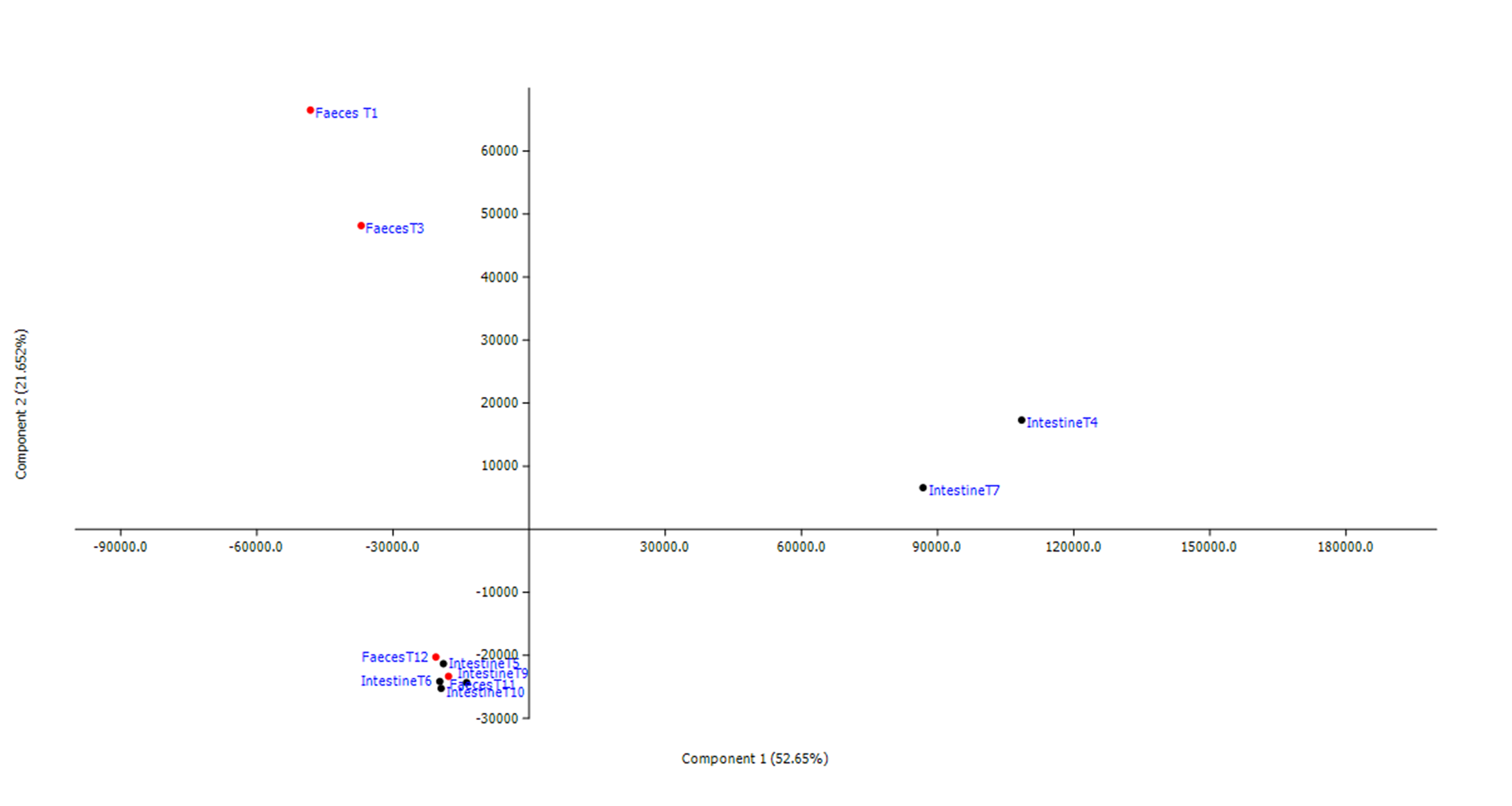

Supplement: Figure S2 — Principal Component Analysis of OTU abundance in each sample. The percentage of variance displayed by the first two components is reported. [file Image2.TIF]
