# Supplementary material for: A First Insight into the Gut Microbiota of the Sea Turtle Caretta caretta
Source: Front Microbiol. 2016 Jul 7;7:1060. doi: 10.3389/fmicb.2016.01060 (PMC4935691; doi:10.3389/fmicb.2016.01060)
Supplement: Table S2 — Results of SIMPER analysis on taxa occurrence. The taxonomic attribution of OTUs, the percentage of contribution and the cumulative contribution are reported. [file Table2.DOCX]

Table S2. Results of SIMPER analysis on taxa occurrence. The taxonomic attribution of OTUs, the percentage of contribution and the cumulative contribution are reported.

1. **T1/T3 samples vs. others***

| **Taxon** | **Phylum** | **Class** | **Order** | **Family** | **Genus** | **Av. dissim** | **Contrib. %** |
| --- | --- | --- | --- | --- | --- | --- | --- |
| **OTU_3** | *Firmicutes* | *Clostridia* | *Clostridiales* | *Peptostreptococcaceae* | *Clostridium XI* | 10.02 | 10.65 |
| **OTU_5** | *Firmicutes* | *Clostridia* | *Clostridiales* | *Clostridiaceae 1* | *Clostridium sensu stricto* | 9.803 | 10.42 |
| **OTU_4** | *Firmicutes* | *Clostridia* | *Clostridiales* | *Peptostreptococcaceae* | *Clostridium XI* | 9.501 | 10.1 |
| **OTU_7** | *Proteobacteria* | *Gammaproteobacteria* | *Oceanospirillales* | *Oceanospirillaceae* | *Oleibacter* | 8.498 | 9.032 |
| **OTU_6** | *Firmicutes* | *Clostridia* | *Clostridiales* | *Defluviitaleaceae* | *Defluviitalea* | 6.369 | 6.769 |
| **OTU_1** | *Firmicutes* | *Bacilli* | *Lactobacillales* | *Enterococcaceae* | *Vagococcus* | 5.824 | 6.19 |

| **Taxon** | **Phylum** | **Class** | **Order** | **Family** | **Genus** | **Av. dissim** | **Contrib. %** |
| --- | --- | --- | --- | --- | --- | --- | --- |
| **OTU_1** | *Firmicutes* | *Bacilli* | *Lactobacillales* | *Enterococcaceae* | *Vagococcus* | 12.25 | 14.17 |
| **OTU_2** | *Firmicutes* | *Clostridia* | *Clostridiales* | *Lachnospiraceae* | *Robinsoniella* | 6.288 | 7.273 |
| **OTU_3** | *Firmicutes* | *Clostridia* | *Clostridiales* | *Peptostreptococcaceae* | *Clostridium XI* | 5.545 | 6.414 |
| **OTU_5** | *Firmicutes* | *Clostridia* | *Clostridiales* | *Clostridiaceae 1* | *Clostridium sensu stricto* | 4.63 | 5.356 |
| **OTU_4** | *Firmicutes* | *Clostridia* | *Clostridiales* | *Peptostreptococcaceae* | *Clostridium XI* | 4.55 | 5.263 |
| **OTU_6** | *Firmicutes* | *Clostridia* | *Clostridiales* | *Defluviitaleaceae* | *Defluviitalea* | 3.124 | 3.614 |
| **OTU_14** | *Bacteroidetes* | *Bacteroidia* | *Bacteroidales* | *Rikenellaceae* | *Rikenella* | 2.949 | 3.411 |
| **OTU_10** | *Firmicutes* | *Clostridia* | *Clostridiales* | *Lachnospiraceae* | Clostridium XlVa | 2.937 | 3.398 |
| **OTU_32** | *Proteobacteria* | *Gammaproteobacteria* | *Enterobacteriales* | *Enterobacteriaceae* | *Buttiauxella* | 2.894 | 3.347 |
| **OTU_28** | *Bacteroidetes* | *Bacteroidia* | *Bacteroidales* | *Bacteroidaceae* | *Bacteroides* | 2.767 | 3.201 |
| **OTU_26** | *Spirochaetes* | *Spirochaetia* | *Spirochaetales* | *Spirochaetaceae* | *Treponema* | 2.693 | 3.115 |

* the first six OTUs are reported which account for more than 50% of total variance
